# Predicting psoriasis using routine laboratory tests with random forest

**DOI:** 10.1371/journal.pone.0258768

**Published:** 2021-10-19

**Authors:** Jing Zhou, Yuzhen Li, Xuan Guo

**Affiliations:** 1 Department of Dermatology, Second Affiliated Hospital of Harbin Medical University, Harbin, PR China; 2 Department of Computer Science and Engineering, University of North Texas, Denton, Texas, United States of America; Cardiff University, UNITED KINGDOM

## Abstract

Psoriasis is a chronic inflammatory skin disease that affects approximately 125 million people worldwide. It has significant impacts on both physical and emotional health-related quality of life comparable to other major illnesses. Accurately prediction of psoriasis using biomarkers from routine laboratory tests has important practical values. Our goal is to derive a powerful predictive model for psoriasis disease based on only routine hospital tests. We collected a data set including 466 psoriasis patients and 520 healthy controls with 81 variables from only laboratory routine tests, such as age, total cholesterol, HDL cholesterol, blood pressure, albumin, and platelet distribution width. In this study, Boruta feature selection method was applied to select the most relevant features, with which a Random Forest model was constructed. The model was tested with 30 repetitions of 10-fold cross-validation. Our classification model yielded an average accuracy of 86.9%. 26 notable features were selected by Boruta, among which 15 features are confirmed from previous studies, and the rest are worth further investigations. The experimental results demonstrate that the machine learning approach has good potential in predictive modeling for the psoriasis disease given the information only from routine hospital tests.

## 1. Introduction

Psoriasis is a systemic, immunological, genetic, chronic inflammatory disease manifesting in the skin or joints. It is characterized by disfigurement and a long clinical course with remissions and relapses, adversely affecting the quality of patients’ life. Its prevalence varies widely, from 0.09% to 11.4%, relying on the studied areas [1]. Until now, its cause is still unclear and conflicting. However, most scholars recognize immunology and genetics play crucial impacts in its pathogenesis [2]. The skin is the most mainly involved, but it is now widely presumed that psoriasis is a multisystemic disorder, frequently accompanied by comorbidities, including cardiac, renal, and metabolic manifestations [3]. Therefore, practical and reliable biomarkers that can indicate psoriasis activity and the potentiality of systemic comorbidities will greatly help reduce variation in diagnosis, improve the outcomes of treatment for patients, and reduce the workload for clinicians. In this study, we proposed a procedure for predicting whether a patient is with or without psoriasis using routine laboratory tests, which can then lead to efficient and repeatable psoriasis diagnosis.

Computer-aided methods have applied to psoriasis study for a number of decades [4]. For example, image-based methods were proposed to evaluate the severity of scaling in psoriasis lesion automatically [5, 6]. Grabe et al. designed a computational model that turns healthy in silico epidermis into one with four central properties of the psoriatic epidermis [7]. Coin et al. developed an exome sequencing pipeline for identifying and genotyping common copy number variations associated with psoriasis [8]. Swindell et al. used *in silico* analysis of transcription factor binding sites for Psoriasis drug development and genome-wide association study interpretation [9]. Sonkoly et al. identify and characterize psoriasis susceptibility-related noncoding RNA gen-based on *in silico* structural and homology studies [10]. As for potential biomarkers for psoriasis diagnosis, no biomarker from routine laboratory tests has been widely used in current studies. Hence, it is interesting to know whether the information from routine laboratory tests can be used as a reflection of the activity of psoriasis.

In this paper, we attempt to determine the association between 81 biomarkers from routine laboratory tests and psoriasis. The data we used is from a population-based hospitalization database. To identify predictors and classify patients, we designed a framework based on Random Forest [11], which is a machine learning method that ensemble a set of decision trees to make prediction and identify important predictors. We identify 26 laboratory parameters that contribute most to the psoriasis classification that yield an average accuracy of 86.9% from 30 repetitions of 10-fold cross-validation. We conclude that information on routine laboratory tests can be used to predict psoriasis status accurately among hospitalized patients.

## 2. Materials and methods

**Fig 1:**
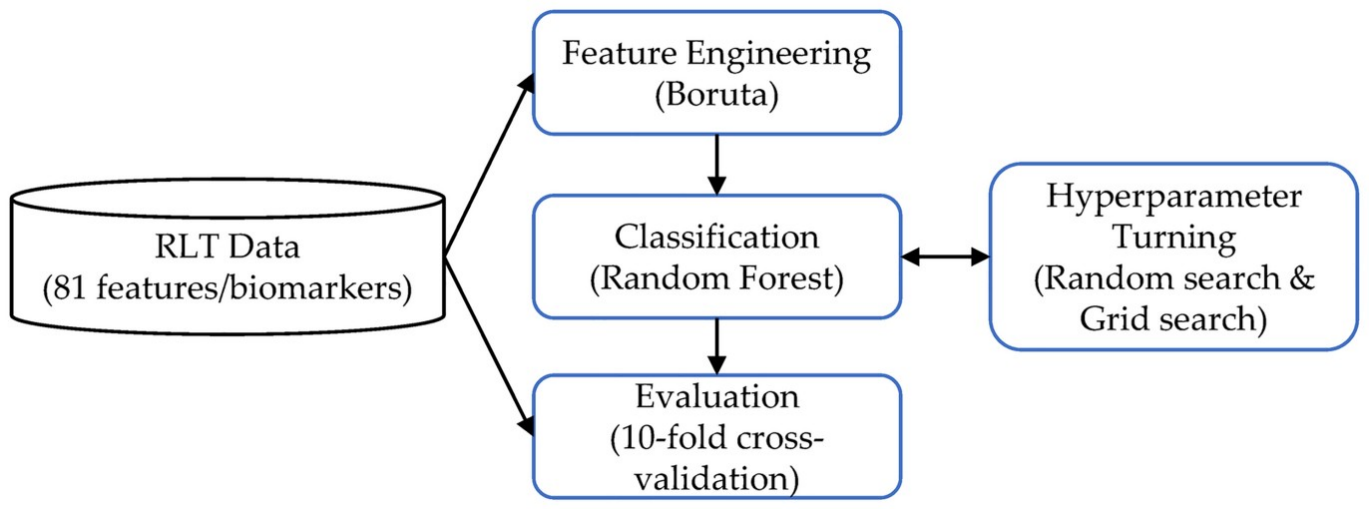
Schematic overview of the proposed methodology.

This section begins with an overview of the proposed predictive modeling pipeline followed by the details for each component. The predictive modeling pipeline consists of four modules, as shown in Fig 1. In the feature engineering module, biomarkers from routine laboratory tests (RLT) are converted into a feature table and a target label vector which are used for training and testing the predictive model. In the classification module, the training data is used to construct the candidate predictive models. The evaluation module examines the results to characterize the learned predictive model. In hyperparameter turning, a grid search is used to generate different candidate model architectures.

### 2.1 Experimental data

#### 2.1.1 Study population

There were 466 psoriasis patients and 520 healthy controls without psoriasis participating in the study from January 2016 to December 2019. Table 1 presents the demographic characteristics of the psoriasis patients and healthy controls, respectively. Psoriasis patients were recruited in the Department of Dermatology of the Second Affiliated Hospital of Harbin Medical University in China. They received skin examinations by two dependent dermatologists, who confirmed the diagnosis of psoriasis. All controls with no history of psoriasis were recruited in the medical check-up center of this hospital. The study population was of Northeast Han Chinese ancestry, living in Northeastern China, and the collected samples can be considered representative of Northeast Han Chinese. Demographic, clinical, and laboratory data were collected from the hospital database. Blood sampling was done at the first visit and was measured using an autoanalyzer (Beckman, Coulter LH 750, America; Tokyo, Modular Analytics, Japan; Sysmex, LF-1000i, Japan).

**Table 1:** Demographic characteristics of 920 participants.

Variables	Values	Case(*n*)	Control(*n*)
Gender	Male	273	265
	Female	193	255
Age in years	1–19	75	37
	20–29	81	80
	30–39	82	106
	40–49	88	131
	50–59	71	93
	60–69	48	60
	70–79	15	12
	80–89	4	1
	90–100	1	0

This study was approved by the ethical committee of the Second Affiliated Hospital of Harbin Medical University. All participants (minors’ parents or guardians) were informed about the screening activities and authorized their or their child(ren)’s participation by signing the appropriate informed consent paperwork. The collected laboratory test results were deidentified to protect patients’ privacy and anonymity.

#### 2.2.2 Excluding criteria

Patients are excluded with the following diseases: other chronic autoimmune/inflammatory diseases, such as RA and inflammatory bowel diseases, hypertension, diabetes, cardiovascular disorders, cancers, overt infections, hematological diseases, chronic liver or kidney diseases, autoimmune disorders, genetic related, and bone marrow transplantation.

#### 2.2.3 Data preprocessing

The sample size in this study is 986 individuals, with 466 psoriasis patients and 520 people without psoriasis. For patients with multiple records, we keep the earliest laboratory test results. In total, we collected 81 features (see Table 1 in S1 File). The percentage of missing values for any patient is less than 5%. For the missing values of the categorical data type, we replaced them with the most frequent values. For the missing values of continuous data type, we replaced them with the average. Categorical values were mapped to integer values using one-hot encoding. Continuous variables were first z-transformed, and their ranges were adjusted to have mean, *μ* = 0, and variance, *σ* = 1. Here, we did not perform any dimension reduction, like Principal Component Analysis (PCA) [12], before feature engineering because Random Forest itself already performs a good regularization without assuming linearity. Summarized clinical information for the study population is shown in Table 2 in S1 File.

### 2.2 Statistical analysis

A set of predictors, including 81 biomarkers from routine laboratory tests, were used in the classification of patients with or without psoriasis. In this study, we use predictors and features interchangeably. We built a prediction procedure based on Random Forests to identify and validate important predictors regarding psoriasis prediction and to indicate patients at high risk of psoriasis. Random forests are ensemble learning method that operate by constructing multiple classification trees for prediction. A single classification tree is a nonparametric, hierarchical classification method that uses recursive splitting to cluster patients that are growingly homogenous with respect to the outcome of interest. For instance, the patients are split into 2 groups based on the biomarker that best separates patients at high risk of psoriasis from those at low risk of psoriasis. The similar process is repeated for each produced group until either all patients are correctly classified, or sufficient homogeneity of resultant groups are met. The classification tree can identify those independent variables that best separate patients as important predictors. Random Forest was originally proposed by Breiman and Cutler [11]. Random forests combine hundreds of classification trees to increase the overall result. Each tree is developed by bagging and feature randomness to create an uncorrelated forest of trees whose prediction by committee is more accurate than that of any individual tree. Our analyses were performed using the Random Forest as given in the scikit-learn library (available at https://scikit-learn.org).

### 2.3 Feature selection and hyperparameter turning

**Fig 2:**
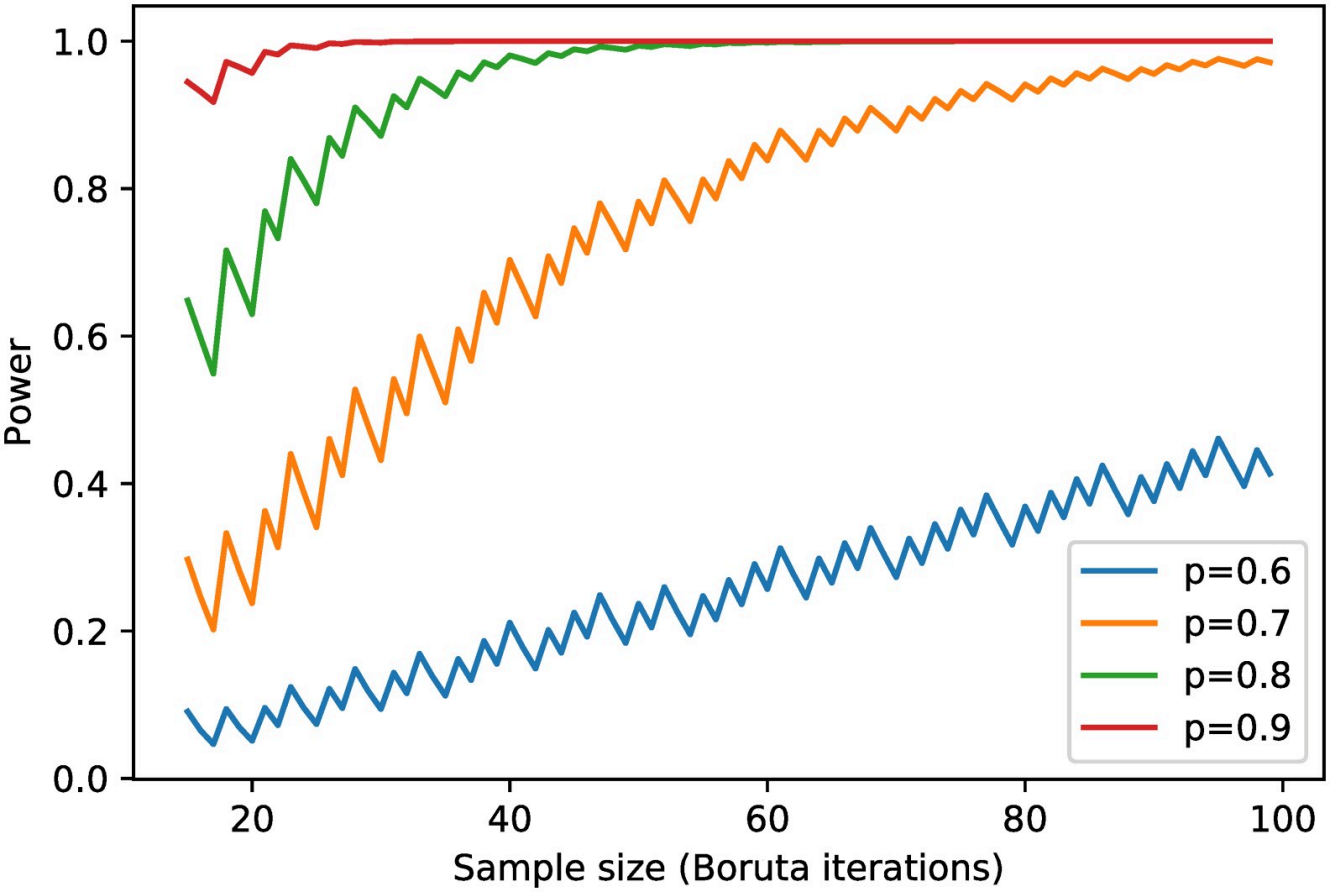
Power curves for the feature selection based on Boruta algorithm.

Feature selection or feature engineering is an essential step in applying machine learning methods. Usually, not all predictors are relevant to the outcome of interest, and their relevance is unknown ahead in time. Many machine learning methods drop in prediction accuracy when the number of predictors is significantly higher than optimal [13]. One of the feature selection methods is the wrapper method in which a subset of features is used to train the model, and features from the subset will be dropped or kept based on the inferences that are drawn from the previous model. In this study, we implement Boruta [14], one of the wrapper methods that finds the importance of a feature. The relative importance of a feature is measured by "Gini importance" or "mean decrease impurity," which is defined as the total decrease in node impurity weighted by the probability of reaching that node averaged over all trees. Boruta algorithm, firstly, creates shadow features to add randomness to the given dataset by creating shuffled copies of all features. It then trains a Random Forest classifier on the extended dataset and measure the Gini importance as defined earlier to evaluate the importance of each feature. It constantly removes real features that are deemed significantly unimportant by checking whether they have higher importance than all shadow features. Finally, Boruta stops when it confirms or rejects all features, or it reaches a specified limit number of iterations. We did a power analysis for the feature selection process. In our Boruta implementation, the importance of features was validated by comparing them with random shuffled shadow features. This is done by simply comparing the number of times a feature did better with the shadow features using a binomial distribution. For an unimportant feature, the binomial probability should be 0.5, which is our null hypothesis, i.e., *H*_0_: *p* = 0.5. The alternative hypothesis is *H*_*a*_: *p* > 0.5. We showed the power curves in Fig 2 with binomial probabilities set to 0.6, 0.7, 0.8, and 0.9, significance level at 3%, and Boruta iterations varied from 15 to 100. In this study, Boruta stopped before reached 100 iterations. We did not perform power analysis for Random Forest prediction because power analysis refers to calculating the power of a hypothesis test, and Random Forest makes no hypothesis.

We employed the 10-fold cross-validation [15] and applied Boruta in each fold. The features deemed as important from all 10 folds were used to tune the hyperparameters of Random Forest. The hyperparameters of Random Forests that we fine-tuned include the number of classification trees in the forest, the number of features considered by each tree when splitting a node, the maximum number of levels in each classification tree, the minimum number of data points placed in a node before the node is split, the minimum number of data points allowed in a leaf node, and the methods for sampling data points. We first used a random search [16] to narrow down the range for each hyperparameter and then explicitly specify every combination of settings with a grid search [16].

## 3. Results

Using the procedures described in the feature selection section, 26 features (biomarkers) were deemed as significantly relevant to the outcome. The 10-fold cross-validation was repeated 30 times to collect the average and the standard deviation of the feature importance of these 26 features. The feature importance is presented in Table 2.

**Table 2:** Feature importance of 26 selected features using Boruta with random forests.

Feature Name	Mean Feature Importance (Standard deviation)	Feature Name	Mean Feature Importance (Standard deviation)
High-density Lipoprotein Cholesterol	0.104 (0.0015)	Low-density Lipoprotein cholesterol (LDL-C)	0.031 (0.0005)
Pre-Albumin	0.08 (0.0010)	Monocyte Count (MONO#)	0.03 (0.0005)
Cystatin-C	0.074 (0.0008)	Urea	0.03 (0.0004)
Total Cholesterol	0.059 (0.0008)	Serum Calcium (Ca)	0.03 (0.0004)
Mean Platelet Volume	0.049 (0.0009)	Urine Color (COL)	0.026 (0.0004)
Blood Pressure	0.043 (0.0006)	Platelet Larger Cell Ratio (P-LCR)	0.025 (0.0005)
Albumin	0.043 (0.0009)	Inorganic Phosphate (P)	0.025 (0.0003)
Platelet Distribution Width	0.042 (0.0009)	Monocyte Ratio (cytes%)	0.024 (0.0004)
Standard Deviation in Red blood Cell Volume Distribution Width	0.038 (0.0007)	Age	0.023 (0.0004)
Triglyceride	0.037 (0.0006)	Urea/Crea(U/C)	0.022 (0.0003)
Total Protein	0.036 (0.0007)	Red Blood Cell Count (RBC)	0.02 (0.0003)
Chloride Ion	0.035 (0.0006)	Cholinesterase (CHE)	0.02 (0.0003)
Serum Magnesium	0.034 (0.0005)	Basophil count (BASO#)	0.019 (0.0004)

The Random Forest model with the best classification performance by hyperparameter turning has the following configuration: the number of trees is set to 500; the number of features to consider when looking for the best split is set to 5; the minimum number of samples required to be at a leaf node is set to 1; the minimum number of samples required to split an internal node is set to 0.

**Fig 3:**
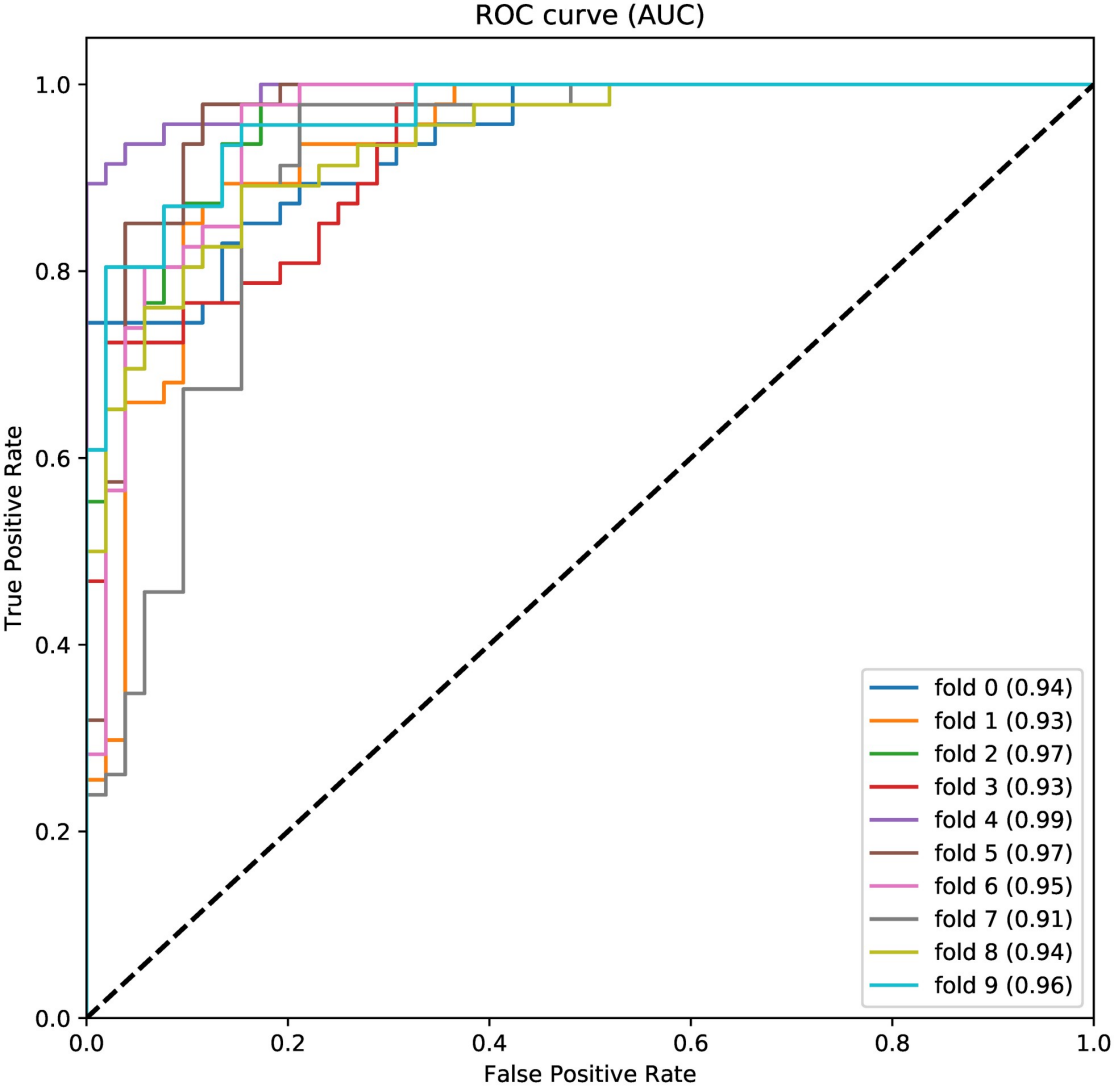
ROC curves for one 10-fold cross-validation using 26 selected features.

We plot ROC (receiver operating characteristic) curves (Fig 3) from one 10-fold cross-validation using the 26 selected features. The ROC curve shows the relationship between sensitivity and specificity for every possible cut-off. The ROC curves from all 10 folds are toward the right up corner, and the average AUC (area under curve) across 10 folds is 0.95. This demonstrates that the learned Random Forest model is efficient in testing the psoriasis status.

The tabulated confusion matrix across all 30 repetitions of 10-folds is presented in Table 3. The metrics are defined as follows:

$$\text{Accuracy}=\frac{TP+TN}{TP+FP+TN+FN}$$

$$\text{Precision}\left(\text{PPV}\right)=\frac{TP}{TP+FP}$$

$$\text{Sensitivity}=\frac{TP}{TP+TN}$$

$$\text{Specificity}=\frac{TN}{FP+TN}$$

where *TP* is the number of individuals correctly predicted to has the disease; *FP* is the number of individuals incorrectly predicted to has the disease; *TN* is the number of individuals correctly predicted not having the disease; *FN* is the number of individuals incorrectly predicted not having the disease. From the results in Table 3 and Fig 3, we conclude that the final Random Forest model well captures the relation between psoriasis status and 26 biomarkers that are commonly used in laboratory tests.

**Table 3:** The overall classification results across all 30 repetitions of 10-folds.

TP	TN	FP	FN	Accuracy (%) (Std)	PPV (%) (Std)	Sensitivity (%) (Std)	Specificity (%) (Std)
410	447	73	56	86.90 (0.003)	84.87 (0.005)	87.98 (0.005)	85.94 (0.005)

## 4. Discussion and conclusions

Since no clinically useful biomarker for psoriasis and its comorbidities has been established so far, we examined 81 biomarkers from routine laboratory tests, and 26 of them were deemed as significantly relevant to psoriasis, based on the procedure described in feature selection section. Among the 26 selected factors, 15 of them align with previous biological studies [1, 17–20], including high-density lipoprotein cholesterol, total cholesterol, mean platelet volume, blood pressure, albumin, platelet distribution width, standard deviation in red blood cell volume distribution width, triglyceride, total protein, serum magnesium, low-density lipoprotein cholesterol, serum calcium, inorganic phosphate, red blood cell count, and urea. In the following, we discuss the relation between these 26 selected biomarkers and psoriasis based on the existing studies.

The biomarkers involving lipidemia are reported to be abnormal in psoriasis compared with healthy controls [17]. Such biomarkers include lower high-density lipoprotein cholesterol, higher total cholesterol, higher blood pressure, higher triglyceride, higher low-density lipoprotein cholesterol in psoriasis patients. These biomarkers usually connect to metabolic syndrome, cardiovascular disease, hypertension, and atherosclerosis, which are the most common comorbidities of psoriasis. As an anti-atherogenic property, high-density lipoprotein cholesterol blocks thrombosis in blood vessels and helps keep the endothelial function [17, 21]. The serum levels are inversely related to stroke risk [22, 23]. No matter psoriasis contributes to symptoms associated with hyperlipidemia, or cardiovascular disease biomarkers increases the risk of developing psoriasis is unclear yet [18–20], abnormal lipidemia may be an important link between psoriasis and metabolic syndrome. So, we should be aware of potential psoriasis conditions for patients with dyslipidemia.

As psoriasis is a chronic inflammatory disease, the studies about the pathogenesis of psoriasis have focused on inflammation. Systemic inflammation has frequently been found in metabolic and cardiovascular diseases [24]. In this study, we found three inflammation-related biomarkers, i.e., mean platelet volume (MPV), platelet distribution width (PDW) and monocyte count. PDW and MPV, as platelet activation markers, were shown significantly higher in patients with psoriasis than controls in recent studies [1, 25]. Platelet activation is believed to play an important role in the immune inflammation. MPV was found obviously increased along with the severity and duration of the psoriasis [1]. Also, MPV levels are associated with various diseases, such as cardiovascular disease and peripheral artery disease [25]. Activated platelet may become common in the inflammatory reaction in psoriasis, although it is unclear whether activated platelets are the cause or the result of psoriasis and its comorbidities inflammation. Our results also suggested that circulating Red Blood Cell counts (RBC) is associated with psoriasis. The studies [3, 26] claimed that the reduction of circulating RBCs in psoriasis patients might be caused by the erythrocyte damage/aging and the accelerated removal process due to inflammatory reaction.

Several studies have demonstrated that total protein and serum albumin levels were significantly decreased among psoriasis patients compared to controls [27, 28], which is agreed with our findings. Many reasons were believed to lead to the changes in total protein and serum albumin. For example, the low albumin level in psoriasis patients is due to scales losing in the extensive lesion and increased turnover rates of keratinocytes [27]. Moreover, the low albumin level results from the increased endogenous catabolism by the liver and splenic dysfunction [27]. The further biological study is needed to verify the mechanism of serum albumin changes in psoriasis. As about 45% of serum calcium connects to albumin, patients with low serum albumin level may show changes in serum calcium level. The close relation between psoriasis and serum calcium has been found in many other studies as well. Because ionic calcium can regulate proliferation and differentiation of keratinocytes, the low calcium level will aggravate psoriasis lesions. Clinically, oral calcium, vitamin D, and topical 1,25-Dihydroxyvitamin D are often used to mitigate psoriasis lesions [29]. The other three biomarkers, including urea, urea creatinine ratio, and phosphate, which were found in our experiments are also supported by the previous study. It has been argued that immunoinflammatory mechanism, such as TH-cell activation, is a common mechanism in psoriasis and renal dysfunction [30]. This may explain why the renal problem is prevalent in psoriasis patients. These biomarkers highlight the potential connection between renal failure and psoriasis.

In this study, psoriasis was found the first time related to 11 of those 26 biomarkers, including pre-albumin, cystatin-c, chloride ion, cholinesterase, basophil count, monocyte count, monocyte ratio, urine color, urea creatinine ratio, age, and platelet-large cell ratio. Further investigations about these 11 biomarkers are needed. Some biomarkers that were found related to Psoriasis in other studies [26, 27, 31–33] were not confirmed by our method. These biomarkers include white blood cell count, neutrophil ratio, neutrophil count, globulin, and fast glucose level. It may be the result of the relatively small sample size, ethnic diversity, and applied diagnostic tools compared to other studies.

## Supporting information

S1 File Appendix. (PDF)

S2 File Data set of 466 psoriasis patients. (XLSX)

S3 File Data set of 520 healthy controls. (XLSX)
