# Supplementary material for: Predicting psoriasis using routine laboratory tests with random forest
Source: PLoS One. 2021 Oct 19;16(10):e0258768. doi: 10.1371/journal.pone.0258768 (PMC8525763; doi:10.1371/journal.pone.0258768)
Supplement: S1 File — (PDF) [file pone.0258768.s001.pdf]

APPENDIX

Table 1. Feature List.

1	Gender	28	Platelet larger cell ratio	55	Inorganic phosphate
2	Age	29	Alanine amiotransferase	56	Serum magnesium
3	Systolic Blood Pressure	30	Aspartate aminotransferase	57	Anion gap
4	Diastolic Blood Pressure	31	AST ALT ratio	58	Total cholesterol
5	White blood cell count	32	Gamma-glutamyl transpeptadase	59	Triglyceride
6	Neutrophil ratio	33	Alkaline phosphatase	60	High-density lipoprotein cholesterol
7	Lymphocyte ratio	34	Total biliary acid	61	Low-density lipoprotein cholesterol
8	Monocyte ratio	35	Pre albumin	62	Urine color
9	Eosinophil ratio	36	total protein	63	Turbidity
10	Basophil ratio	37	Albumin	64	Urine specific gravity
11	Neutrophil count	38	Globulin	65	Urine potential of hydrogen
12	Lymphocyte count	39	Albumin globulin ratio	66	Urine leucocyte
13	Monocyte count	40	Total bilirubin	67	Urine nitrite
14	Eosinophil count	41	Direct bilirubin	68	Urine protein
15	Basophil count	42	Indirect bilirubin	69	Urine glucose
16	Hemoglobin	43	Cholinesterase	70	Urine ketone
17	Red blood cell count	44	Cystatin-C	71	Urobilinogen
18	Hematocrit	45	Urea	72	Urine occult blood
19	Mean corpuscular volume	46	Creatinine	73	Urine red blood cell
20	Mean corpuscular hemoglobin	47	Urea/Crea ratio	74	Urine leucocyte
21	Mean corpuscular hemoglobin concentration	48	Uric acid	75	Epithelial cell count
22	Coefficient variation of red blood cell volume distribution width	49	Bicarbonate	76	Yeast-Like Cells
23	Standard deviation in red cell distribution width	50	Glucose	77	Urine crystallization
24	Platelet count	51	Potassium ion	78	Red blood cell
25	Mean platelet volume	52	Sodium ion	79	White blood cell
26	Platelet distribution width	53	Chloride ion	80	Epithelial cells
27	Thrombocytocrit	54	Serum calcium	81	Cast

Table 2. Summarised biological and clinical information for the study population.

(A) Continuous features	Psoriasis patients (mean ± std. dev.)	Healthy controls (mean ± std. dev.)
Age	39.17 ± 18.23	42.10 ± 14.38
Systolic Blood Pressure	120.24 ± 18.00	123.46 ± 12.96
Diastolic Blood pressure	79.35 ± 11.65	79.42 ± 8.29
White blood cell count (WBC)	7.27 ± 3.21	6.48 ± 1.34
Neutrophil ratio (NEUT%)	58.55 ± 11.38	58.59 ± 20.88
Lymphocyte ratio (LYMPH%)	31.75 ± 10.68	33.81 ± 7.35
Monocyte ratio (cytes%)	6.60 ± 4.81	5.70 ± 1.58
Eosinophil ratio (EO)	2.89 ± 2.54	2.31 ± 1.97
Basophil ratio (BASO%)	0.27 ± 0.24	0.33 ± 0.45
Neutrophil count (NEUT#)	4.46 ± 3.01	3.81 ± 1.60
Lymphocyte count (LYMPH#)	2.63 ± 10.32	2.16 ± 0.57
Monocyte count (MONO#)	0.45 ± 0.21	0.37 ± 0.19
Eosinophil count (EO#)	0.20 ± 0.18	0.15 ± 0.15
Basophil cout (BASO#)	0.02 ± 0.05	0.02 ± 0.06
Hemoglobin (HGB)	137.62 ± 17.03	143.24 ± 13.88
Red blood cell count (RBC)	4.58 ± 2.22	4.73 ± 0.52
Hematocrit (HCT)	41.21 ± 4.73	42.92 ± 4.93
Mean corpuscular volume (MCV)	91.97 ± 6.25	109.43 ± 418.97
Mean corpuscular hemoglobin (MCH)	30.74 ± 2.05	30.20 ± 2.30
Mean corpuscular hemoglobin concentration (MCHC)	332.28 ± 22.91	333.09 ± 16.30
Coefficient variation of red blood cell volume distribution width (RDW-CV)	13.21 ± 1.26	12.97 ± 1.09
Standard deviation in red cell distribution width (RDW-SD)	44.23 ± 5.10	42.91 ± 2.70
Platelet count (PLT)	245.86 ± 78.01	236.22 ± 51.94
Mean platelet volume (MPV)	10.91 ± 4.52	9.92 ± 1.69
Platelet distribution width (PDW)	13.08 ± 5.79	14.15 ± 2.43
Thrombocytocrit (PCT)	0.26 ± 0.08	0.36 ± 1.96
Platelet larger cell ratio (P-LCR)	30.92 ± 8.15	31.76 ± 7.92
Alanine amiotransferase (ALT)	21.62 ± 21.25	18.31 ± 9.15
Aspartate aminotransferase (AST)	20.48 ± 10.89	18.28 ± 4.79
AST/ALT	1.21 ± 0.67	1.37 ± 4.15
γ-glutamyl transpeptadase (GGT)	33.00 ± 43.56	24.08 ± 20.69
Alkaline phosphatase (ALP)	85.89 ± 45.55	70.05 ± 23.22
Total biliary acid (TBA)	4.67 ± 6.12	3.70 ± 5.34
Pre albumin (PA)	229.35 ± 76.55	282.41 ± 63.87
Total protein (TP)	67.93 ± 6.05	71.79 ± 5.35
Albumin (ALB)	41.97 ± 5.03	45.00 ± 3.93
Globulin (GLO)	26.35 ± 9.39	26.71 ± 4.14
Albumin/Globulin	1.68 ± 0.33	1.76 ± 0.72
Total bilirubin (TBIL)	10.69 ± 7.09	11.46 ± 5.57
Direct bilirubin (DBIL)	4.20 ± 3.36	4.14 ± 1.40
Indirect bilirubin (IDBIL)	6.37 ± 4.36	7.32 ± 4.35
Cholinesterase (CHE)	7885.17 ± 2048.02	8737.74 ± 1742.42
Cystatin-C	0.72 ± 0.30	0.74 ± 3.10
Urea	4.56 ± 1.58	5.06 ± 1.40
Creatinine	66.43 ± 25.36	66.55 ± 15.13
Urea/Crea	87.34 ± 323.26	77.59 ± 24.27
Uric acid (UA)	315.44 ± 138.35	313.93 ± 84.59
Bicarbonate (HCO3)	26.93 ± 14.65	26.51 ± 2.15

Glucose	6.77 ± 29.15	5.31 ± 2.06
Potassium ion	5.68 ± 20.91	4.32 ± 0.39
Sodium ion (Na)	141.28 ± 3.57	141.37 ± 2.01
Chloride ion (Cl)	105.05 ± 7.61	104.41 ± 5.63
Serum calcium (Ca)	2.82 ± 10.99	2.29 ± 0.12
Inorganic phosphate (P)	1.26 ± 0.56	1.37 ± 5.24
Serum magnesium (Mg)	0.98 ± 0.94	1.35 ± 6.39
Anion gap (AG)	14.37 ± 6.09	18.43 ± 71.76
Total cholesterol (T-CH)	5.31 ± 22.84	6.09 ± 29.98
Triglyceride	1.54 ± 1.05	1.31 ± 0.90
High-density lipoprotein cholesterol (HDL-C)	1.23 ± 0.37	1.41 ± 0.31
low-density lipoprotein cholesterol (LDL-C)	2.68 ± 0.76	2.80 ± 1.21
Urine specific gravity (SG)	1.02 ± 0.01	3.17 ± 46.39
Urine potential of hydrogen (U-PH)	6.02 ± 0.78	6.23 ± 2.33
Urine red blood cell (RBC)	31.14 ± 265.26	9.94 ± 41.60
Urine leucocyte (WBC)	29.21 ± 137.49	9.30 ± 33.06
Epithelial cell count (EC)	6.60 ± 15.09	5.78 ± 12.20
Red blood cell (RBCH)	2.84 ± 13.39	1.72 ± 7.46
White blood cell (WBCH)	2.62 ± 8.41	1.59 ± 5.98
Epithelial cells (ECH)	1.06 ± 2.54	0.96 ± 2.25
Cast (CASTL)	0.00 ± 0.00	0.08 ± 0.82

(B) Categorial features	Psoriasis patients (value: frequency)	Healthy controls (value: frequency)
Gender	Male: 273<br>Female: 193	Male: 265<br>Female: 255
Urine color	Yellow: 297<br>Faint yellow: 108<br>Clear: 53<br>NA: 8	Yellow: 420<br>Faint yellow: 45<br>Clear: 37<br>NA: 18
Turbidity	Turbidity: 46<br>Low turbidity: 150<br>Clear: 262<br>NA: 8	Turbidity: 28<br>Low turbidity: 153<br>Clear: 321<br>NA: 18
Urine leucocyte	Negative: 375<br>+-: 18<br>1: 25<br>2: 22<br>3: 14<br>4: 6<br>NA: 6	Negative: 448<br>+-: 19<br>1: 5<br>2: 10<br>3: 5<br>NA: 17
Urine nitrite	Negative: 450<br>1: 3<br>2: 7<br>NA: 6	Negative: 503<br>2: 1<br>NA: 16
Urine protein	Negative: 345<br>+-: 82<br>1: 28<br>2: 2<br>3: 3<br>NA: 6	Negative: 421<br>+-: 67<br>1: 13<br>2: 1<br>NA: 18
Urine glucose	Normal: 440<br>+-: 6	Normal: 490<br>+-: 4

	1: 2<br>3: 4<br>4: 8<br>NA: 6	1: 2<br>2: 1<br>3: 4<br>4: 3<br>NA: 16
Urine ketone	Negative: 441<br>+-: 10<br>1: 7<br>2: 1<br>3: 1<br>NA: 6	Negative: 494<br>+-: 6<br>1: 1<br>NA: 6
Urbilinogen	Normal: 406<br>+-: 1<br>1: 46<br>2: 6<br>3: 1<br>NA: 6	Normal: 477<br>1: 21<br>2: 1<br>NA: 16
Urine blood	Negative: 430<br>+-: 12<br>1: 10<br>2: 4<br>3: 4<br>NA: 6	Negative: 443<br>+-: 26<br>1: 23<br>2: 1<br>3: 3<br>NA: 17
Yeast-Like Cells	Negative: 460<br>NA: 6	Negative: 500<br>Positive: 4<br>NA: 16
Urine crystallization	Negative: 454<br>Positive: 6<br>NA: 6	Negative: 478<br>Positive: 26<br>NA: 16
